# Haplotype Analysis of Interleukin-8 Gene Polymorphisms in Chronic and Aggressive Periodontitis

**DOI:** 10.1155/2013/342351

**Published:** 2013-12-03

**Authors:** Petra Borilova Linhartova, Jan Vokurka, Hana Poskerova, Antonin Fassmann, Lydie Izakovicova Holla

**Affiliations:** ^1^Department of Pathophysiology, Faculty of Medicine, Masaryk University, 625 00 Brno, Czech Republic; ^2^Clinic of Stomatology, Institutions Shared with St. Anne's Faculty Hospital, Faculty of Medicine, Masaryk University, 656 91 Brno, Czech Republic

## Abstract

*Objectives*. Periodontitis is an inflammatory disease characterized by connective tissue loss and alveolar bone destruction. Interleukin-8 (*IL8*) is important in the regulation of the immune response. The aim of this study was to analyze four polymorphisms in the *IL8* gene in relation to chronic (CP) and aggressive (AgP) periodontitis. *Methods*. A total of 492 unrelated subjects were included in this case-control association study. Genomic DNA of 278 patients with CP, 58 patients with AgP, and 156 controls were genotyped, using the 5′ nuclease TaqMan assay, for *IL8* (rs4073, rs2227307, rs2227306, and rs2227532) gene polymorphisms. Subgingival bacterial colonization was investigated by the DNA-microarray detection kit in a subgroup of subjects (*N* = 247). *Results*. Allele and genotype frequencies of all investigated *IL8* polymorphisms were not significantly different between the subjects with CP and/or AgP and controls (*P* > 0.05). Nevertheless, the A(−251)/T(+396)/T(+781) and T(−251)/G(+396)/C(+781) haplotypes were significantly less frequent in patients with CP (2.0% versus 5.1%, *P* < 0.02, OR = 0.34, 95% CI: 0.15–0.78, resp., 2.0% versus 4.5%, *P* < 0.05, OR = 0.41, 95% CI: 0.18–0.97) than in controls. *Conclusions*. Although none of the investigated SNPs in the *IL8* gene was individually associated with periodontitis, some haplotypes can be protective against CP in the Czech population.

## 1. Introduction 

Periodontitis is an inflammatory disease which is initiated and maintained by the gram-negative bacteria of the subgingival biofilm [1]. Specific pathogen associated molecular patterns (PAMPs) and bacterial virulence factors stimulate an inflammatory host response that finally results in destruction of periodontal tissue and tooth loss [2]. Chronic periodontitis (CP) and generalized aggressive forms of periodontitis (AgP) appear to be associated with certain pathogens, including *Porphyromonas gingivalis*, *Campylobacter rectus*, *Tannerella forsythia*, *Peptostreptococcus micro*, and *Treponema* species [3, 4]. *Treponema denticola*, *P. gingivalis*, and *T. forsythia*, characterized as the “red complex,” were strongly associated with the clinical progression of chronic periodontitis [1–5]. In contrast, AgP was more often diagnosed in patients positive for *Aggregatibacter actinomycetemcomitans*, but there were many individuals with AgP who did not harbor this microorganism [6].

In addition to the microbial challenge, other factors, such as genetics, environment, and host factors, play a role in the pathogenesis of these diseases [7–9]. Various compounds, such as cytokines, representing an important pathway of connective tissue destruction in periodontitis, have been detected in gingival crevicular fluid (GCF). IL-8, a member of the CXC chemokine family, was originally described by Matsushima and Oppenheim [10]. It is the most important chemoattractant and activator of human neutrophils and an important mediator for granulocyte accumulation [11]. IL-8 is involved in the initiation and amplification of acute inflammatory reactions and chronic inflammatory processes. Functions of IL-8 are mediated through two receptors (CXCR1 and CXCR2); the expression was detected on numerous cell lineages, including neutrophils and epithelial cells [12]. Gingival epithelial cells (GEC) are capable of upregulating *IL8* expression rapidly in response to *A. actinomycetemcomitans* challenge, facilitating thus the recruitment of neutrophils as a host defense mechanism [13, 14]. *IL8 *expression in GEC is induced by *P. gingivalis* [15] and *T. forsythia* [16]; IL-8 production by gingival fibroblast cultures is also affected by lipopolysaccharides of *P. gingivalis* and *P. intermedia* [17]. The IL-8 levels in gingival crevicular fluid (GCF) are valuable in detecting the inflammation of periodontal tissue [18–20], and periodontal therapy reduces the IL-8 levels in GCF [21].

IL-8 is encoded by the *IL8* gene located on chromosome 4q13-21 (GenBank accession number M28130.1), consisting of four exons, three introns, and the proximal promoter region [22]. Several polymorphisms have been reported in the *IL8 *gene [23–26] and some of them can regulate the IL-8 production. Some of SNPs in the *IL8* gene such as −845T/C (rs2227532), −738T/C, −251A/T (rs4073, previously referred to as −353A/T), +396T/G (rs2227307), and +781C/T (rs2227306) have been studied in patients with AgP or CP in the Brazilian population [27–32]. In addition, *IL8* −251T allele, which was associated with higher production of IL-8, increased the risk of developing acute suppurative form of apical periodontitis (AP), whereas *IL8* −251A “low-producing” allele was associated with chronic nonsuppurative form of AP in the Colombian population [33]. In the Chinese population, *IL8* −251A allele has been associated with decreased susceptibility to CP [34]. The cross-sectional study in Iran has also focused on the study of polymorphisms in the *IL8* gene but did not specify whether it was for patients with CP or AgP [35]. To date, no study analyzing allele, genotype, or haplotype frequencies of *IL8* gene polymorphisms in patients with periodontitis has been performed in Caucasians.

The aim of this study was to associate four SNPs in the *IL8* gene (rs4073, rs2227307, rs2227306, and rs2227532) and their haplotypes to CP and AgP and subgingival bacterial colonization in the Czech population.

## 2. Material and Methods

The study was performed with the approval of the Committees for Ethics of the Medical Faculty, Masaryk University Brno and St. Anne's Faculty Hospital. Written informed consent was obtained from all participants before inclusion in the study, in line with the Helsinki Declaration.

### 2.1. Study Population

All patients were recruited from the patient pool of the Periodontology Department, Clinic of Stomatology, St. Anne's Faculty Hospital, Brno, from 2005 to 2011. They had at least 20 remaining teeth and were in good general health. Exclusion criteria included history of cardiovascular disorders (such as coronary artery diseases or hypertension), diabetes mellitus, malignant diseases, immunodeficiency, current pregnancy, or lactation. Controls were selected from subjects referring to the Clinic of Stomatology for reasons other than periodontal disease (such as dental caries, orthodontic consultations, preventive dental check-ups, etc.) during the same period as patients and matched for age, gender, and smoking status. Similarly as patients, all controls had at least 20 remaining teeth and were in good general health. Exclusion criteria were the same as those applied for patients with periodontitis.

### 2.2. Case-Control Association Study

A total of 492 unrelated Caucasian subjects of exclusively Czech ethnicity from the region of South Moravia were included in this case-control association study. Diagnosis of nonperiodontitis/periodontitis was based on the detailed clinical examination, medical and dental history, tooth mobility, and radiographic assessment. Probing depth (PD) and attachment loss (CAL) were collected with a UNC-15 periodontal probe from six sites on every tooth present. The loss of the alveolar bone was determined radiographically. We used the index of Mühlemann to evaluate decreases in alveolar bone levels.

1. Generalized CP group (*N* = 278): all patients with chronic periodontitis (CP) fulfilled the diagnostic criteria defined according to CAL levels by the International Workshop for a Classification of Periodontal Diseases and Conditions for Chronic Periodontitis [36]. Inclusion criteria for patients suffering from generalized chronic periodontitis were as follows: ≥30% of the teeth were affected, PD was ≥4 mm, and the amount of CAL was consistent with the presence of dental plaque.
2. Generalized AgP group (*N* = 58): patients with aggressive periodontitis with age at disease onset <35 years, attachment loss of 4 mm or more in at least 30% of the teeth (at least three of the affected teeth were not first molars and incisors), and the severity of attachment loss being inconsistent with the amount of dental plaque were included in this study.
3. Control group (healthy/nonperiodontitis) (*N* = 156): controls were screened using a WHO probe and the CPITN (Community Periodontal Index of Treatment Needs) was assessed [37]; values of the CPITN index in controls were less than 3.

In order to adjust for the effect of smoking history on periodontal disease, the subjects (patients and controls) were classified into the following groups: subjects who never smoked (referred to as nonsmokers) and subjects who were former smokers for ≥5 pack years or current smokers (referred to as smokers). The pack years were calculated by multiplying the number of years of smoking by the average number of cigarette packs smoked per day.

### 2.3. Genetic Analysis

#### 2.3.1. Isolation of Genomic DNA

DNA for genetic analysis was extracted from the peripheral blood leukocytes using standard phenol/chloroform procedures with proteinase K according to Sambrook et al. [38]. Isolation and storage of DNA (working samples at concentrations of 50 ng *μ*L^−1^ at 4°C) as well as the genotyping of samples were conducted in the laboratory of the Department of Pathophysiology, Faculty of Medicine, Masaryk University, Brno, Czech Republic.

#### 2.3.2. SNPs Genotyping TaqMan Assay

Four SNPs (−845C/T rs2227532, −251A/T rs4073, +396G/T rs2227307, and +781C/T rs2227306) in the *IL8* gene were genotyped using the 5′ nuclease TaqMan assay for allelic discrimination. Individual fluorogenic TaqMan probes, consisting of an oligonucleotide labelled with both a fluorescent reporter dye, FAM, and a quencher dye, VIC, were obtained from Life Technologies (Grand Island, NY, USA). Each reaction mixture was prepared using TaqMan Genotyping Master Mix (12.5 *μ*L), TaqMan SNP Genotyping Assay (1.25 *μ*L), and 50 ng of genomic DNA in 17.5 *μ*L of dH_2_O to make a 25.0 *μ*L reaction volume. Genotyping was carried out simultaneously with 88 samples on 96-well plate (+8 negative controls). Details on SNPs detection are summarized in Table 1. The PCR thermocycling protocol consisted of 10 min at 95°C, followed by 40 cycles of 15 s at 92°C and 1 min at 60°C. Each genotyping plate contained eight wells without any DNA template (negative controls) and randomly selected duplicate samples (10% of plate samples). Allele genotyping from fluorescence measurements was then obtained using the ABI PRISM 7000 Sequence Detection System. SDS version 1.2.3 software was used to analyze real-time and endpoint fluorescence data. Genotyping was performed by one investigator (P. B. L.) unaware of the phenotype.

#### 2.3.3. DNA Microarray Analysis of Oral Pathogens

Subgingival bacterial colonization (*Aggregatibacter actinomycetemcomitans*, *Porphyromonas gingivalis*, *Prevotella intermedia*, *Tannerella forsythia*, *Treponema denticola*, *Peptostreptococcus micros*, *and Fusobacterium nucleatum*) in subgingival pockets was investigated by the DNA microarray based on a periodontal pathogen detection kit (Protean Ltd., Ceske Budejovice, CR) in a subgroup of randomly selected subjects (*N* = 151 for CP, *N* = 21 for AgP, and *N* = 75 for controls) before subgingival scaling. Microbial samples were collected from the deepest pocket in periodontitis patients (and from the deepest sulcus in healthy subjects) of each quadrant by inserting a sterile paper point into a base of the pocket for 20 seconds. Bacterial plaque samples from each individual were pooled in one tube. This test determined the individual pathogens semiquantitatively as follows: (−) undetected, which corresponds to the number of bacteria less than 10^3^, (+) slightly positive corresponding to the number of bacteria 10^3^ to 10^4^, (++) positive corresponding to the number of bacteria 10^4^ to 10^5^, and (+++) strongly positive, with the number of bacteria higher than 10^5^.

### 2.4. Statistical Analysis

Comparisons were made between allelic and genotype frequencies in the patients with chronic or aggressive form of periodontitis and control population. The allele frequencies were calculated from the observed numbers of genotypes. The significance of differences in the allele frequencies among groups was determined by Fisher's exact test. *χ* ^2^ analysis was used to test for deviation of genotype distribution from Hardy-Weinberg equilibrium and comparison of differences in genotype combinations among groups.

To examine the linkage disequilibrium (LD) between all SNPs, pairwise LD coefficients (D′) and haplotype frequencies were calculated using the SNP Analyzer 2 program (http://snp.istech.info/istech/board/login_form.jsp). Variations in the quantity of subgingival bacteria corresponding to the particular genotypes/alleles were tested by *χ* ^2^ and Fisher's exact tests. Differences were considered significant at *P* < 0.05.

Power analysis was performed with respect to the case-control design of the study taking the incidence rate of markers and estimate of the odds ratio (OR) as end-point statistical measures. ORs with corresponding 95% confidence intervals (CI) were estimated using logistic regression models, adopting age, sex, and smoking as adjusting covariates. All calculations were performed using Statistica ver. 10.0 (StatSoft Inc., Tulsa, OK, USA) and SPSS software (SPSS 20.0.1, IBM Corporation, 2011).

## 3. Results

### 3.1. Case-Control Study

The mean ages for AgP patients (37.0 ± 8.2; years ± SD) and healthy subjects (41.0 ± 12.3) did not differ between the two groups. However, subjects with CP were significantly older (47.9 ± 8.7) than the patients with AgP (*P* < 0.05). Nearly, 27% of the periodontitis patients (28.0% of CP and 26.0% of AgP) and 28.0% of healthy subjects were smokers. There were no significant differences between the subjects with periodontitis and controls regarding the mean percentage of smokers and ratio of males/females (77/79 in controls, 136/142 in patients with CP, and 25/33 in patients with AgP).

Sample size of the study was planned in standard power calculation for case-control design of the study with the null and alternative hypotheses expressed on the basis of OR. The design was prospectively optimized assuming the prevalence of examined attribute among controls to be 0.5. The recruited sample (278 cases, 156 controls) allowed a statistically significant detection of OR out of the range of 0.55–1.80 (alpha = 0.05, power = 0.80). In case of the AgP group (58 cases, 156 controls), the statistically significantly detectable ORs estimates were out of the range of 0.39–2.59.

### 3.2. Single Nucleotide Polymorphism Analysis

All studied polymorphisms were in the Hardy-Weinberg equilibrium in the control group. Allele and genotype frequencies of all investigated* IL8* polymorphisms were not significantly different between the subjects with CP and/or AgP and controls (*P* > 0.05; Table 2). Considering that in the Czech population, SNP *IL8* −845TT genotype occurred in 98.4% of CP patients and even 100% of controls and AgP patients, we analyzed this polymorphism only in the subgroup of subjects (*N* = 193).

### 3.3. Haplotype Analysis

Based on the previous study [29] the haplotype analysis of these selected SNPs was performed in the *IL8 *gene (−251T/A rs4073, +396T/G rs2227307, and +781C/T rs2227306). All variants in the *IL8* gene were in tight linkage disequilibrium with each other to various degrees (D′ = 0.793–1.000 in controls, D′ = 0.889–0.951 in patients with CP, and D′ = 0.891–1.000 in patients with AgP). The complex analysis revealed differences in *IL8* haplotype frequencies. Specifically, the A(−251)/T(+396)/T(+781) and T(−251)/G(+396)/C(+781) haplotypes were significantly less frequent in patients with CP (2.0% versus 5.1%, resp., 4.5%, *P* < 0.05) (Table 3). The decreased frequency of the TGC haplotype alleles in patients with CP was confirmed by the observation that TGC/TTC haplotype (arranged as genotypes) was less frequent in patients with CP (0.4% versus 2.6%, *P* < 0.05, OR = 0.09, 95% CI = 0.01–0.96). Moreover, an uncommon ATT/ATT haplotype (1.15% of the studied population) was found more, but nonsignificantly, in non-periodontitis controls (2.6% versus 0.4%, *P* = 0.07). There was also a nonsignificant trend in the ATC/TTC haplotype association with CP (2.2% versus 0.0%, *P* = 0.08, Table 4).

### 3.4. Microbiological Analysis

*F. nucleatum* occurred less frequently in nonperiodontitis subjects (*N* = 75) positive for T allele of *IL8* +396G/T variant (49.2% versus 77.8%, *P* < 0.02; OR = 0.28, 95% CI = 0.09–0.89) or TT genotype (21.2% versus 55.6%, *P* < 0.05; OR = 0.22, 95% CI = 0.05–0.91). In contrast, *IL8* −251T allele carriers had an increased OR for individual presence of *A. actinomycetemcomitans* in AgP (*N* = 21) patients (91.7% versus 40.0%, *P* < 0.01; OR = 16.5, 95% CI = 1.88–145.0) and also TT genotype was more often found in *A. actinomycetemcomitans* presence (83.0% versus 13.3%, *P* < 0.01; OR = 32.5, 95% CI = 2.38–443.2). Patients with CP (*N* = 151) carrying CC genotype of *IL8* +781T/C variant had less frequent presence of *T. forsythia* in their subgingival microflora than subjects without this genotype (21.6% versus 35.1%, *P* < 0.05; OR = 0.51, 95% CI = 0.25–1.05). However, the relationship between periodontal bacteria and *IL8* gene polymorphisms must be assessed very carefully regarding small numbers of subjects in the respective subgroups.

## 4. Discussion

Cytokines involved in the inflammatory process, such as *IL8* and their genes, are important potential modifiers of individual susceptibility to AgP or CP. Although none of the investigated SNPs in the *IL8* gene was individually associated with aggressive or chronic periodontitis, the patients with CP showed lower A(−251)/T(+396)/T(+781) and T(−251)/G(+396)/C(+781) haplotype frequencies than the controls. The association of TGC haplotype with CP was confirmed by the relationship between TGC/TTC haplotype (arranged as genotypes) and CP. These results confirm the hypothesis that haplotypes are more powerful for detecting susceptibility alleles than individual polymorphisms. Our results differ from those obtained by Scarel-Caminaga et al. [29] who associated ATC/TTC and AGT/TGC haplotypes with chronic periodontitis in the Brazilian population. These contradictory findings may be due to variability of the examined populations. For example, the frequency of the ATC haplotype in the Czech population was less than 3.4%, compared to 23.7% in the Brazilians [29]. In the Brazilian population, some haplotypes of *IL8* −845(T/C)/−738(T/A)/−353(A/T) variants showed significant association to, or protection against, CP [28]. Of the three *IL8* SNPs, only one polymorphism was the same as in our study (i.e., SNP −251 (rs4073) referred to as −353 in Brazilian study). Polymorphism −845T/C (rs2227532) was also investigated in this study, but regarding a very low frequency of C allele, it was analyzed only in several individuals and therefore it was not included for any further haplotype assessment.

To date, eight studies evaluating the association of *IL8* SNPs (−251, +396, and +781) and CP or AgP in different populations (mostly in Brazilian but none in the Caucasian population) have been performed, with contradictory results [27–32, 34, 35]. Most of these studies analyzed only the individual SNP and were focused on examining of CP; only Andia et al. [32] studied the relationship between *IL8* −251 variant and AgP; however, no association was found. Similarly, Kim et al. [27] failed to find any association between CP and allele or genotype distribution of *IL8* −251 SNP. In contrast, another study discovered a significant association between *IL8* −251 SNP and CP in nonsmokers. The *IL8* −251TA heterozygote genotype was associated with increased levels of *IL8* mRNA transcripts and A allele had an increased risk for developing periodontitis [30]. This is consistent with the observation that the A allele of SNP *IL8* −251 tended to be associated with higher IL-8 production in lipopolysaccharide- (LPS-) stimulated human whole blood [25]. Very recently, Li et al. [34] found that A allele of *IL8* −251 variant was associated with decreased susceptibility to CP in Chinese population. Houshmand et al. [35] found a significant difference in the genotype frequencies of *IL8* −251A/T and +396G/T SNPs between subjects with periodontitis and a control group in Hamedan, Iran, but did not specify whether it was for patients with CP or AgP. Conversely, Corbi et al. [31] showed that the genetic susceptibility to CP in the *IL8* gene was not associated with worse periodontal clinical parameters and increased IL-8 concentration. With the exception of Scarel-Caminaga et al. [29] who associated the +396TT genotype with CP, no association between SNP at position +396 or +781 and CP in the Brazilian population was discovered.

Several studies have examined polymorphisms in interleukins in connection with a subgingival bacterial colonization in patients with periodontitis. In this study, SNPs in the gene encoding IL-8 were associated with the presence of pathogenic bacteria in subgingival dental plaque. The results showed that *IL8* −251T allele carriers had an increased OR for the individual presence of *A. actinomycetemcomitans* in AgP patients (*P* < 0.01) and an increased OR was also found for the presence of *T. forsythia* for T allele of *IL8* +781 in CP patients (*P* < 0.05). These data are in agreement with the notion that individual genetic susceptibility may influence the host response to infection [39]. Nibali et al. [40] found association between *IL6* SNPs and *A. actinomycetemcomitans*, which they confirmed by the haplotype analysis. Specifically, *IL6* −174GG genotype was associated with high (above median) counts of *A. actinomycetemcomitans* (both in all subjects and periodontally healthy subjects only) in Indians [41]. But Nibali et al. [42] also suggested that only a detection of known periodontopathogenic bacteria could not discriminate different forms of periodontitis. In contrast, Schulz et al. [43] found no evidence that SNPs in *IL1* gene cluster could be associated with subgingival colonization with *A. actinomycetemcomitans* and could thus be an independent risk indicator of AgP. In addition, Finoti et al. [44] observed that periodontal destruction may occur in patients who are considered to be genetically susceptible to CP with a lower microbial challenge because of the presence of the *IL8* ATC/TTC haplotype than in patients without this haplotype.

There are some limitations to this study that need to be considered. First, the major complicating factor in the study of isolated locus (such as *IL8*) is the nature of periodontitis as a multifactorial disease in which interaction between multiple genes plays a role and each genetic polymorphism has generally only a small effect. In addition, the interaction of gene variants with environmental factors (such as bacterial pathogens), socioeconomic factors, BMI, and others not analyzed in this study, potentially affect the observed phenotype. Second, the case-control approach used is generally quite vulnerable to the population stratification, for example, due to different ethnic origin. The present sample, however, is exclusively of the Czech Caucasian origin, restricted to the limited geographical area populated by quite homogeneous population with low admixture. Finally, we did not measure RNA expression or protein levels of IL-8, therefore, we do not know the functional consequences of these polymorphisms in our subjects.

In conclusion, although none of the investigated SNPs in the *IL8* gene were individually associated with periodontitis, some haplotypes can be protective against CP in the Czech population. Clinical significance of these findings is low due to a very low frequency of the “protective” haplotypes. The individual *IL8* variants were associated with subgingival colonization with *A. actinomycetemcomitans *in AgP and with *T. forsythia *in CP in the Czech population. However, these relationships must be assessed very carefully regarding small numbers of subjects in the respective subgroups. Further studies are needed to clarify the association of these polymorphisms with periodontal diseases in other populations.

## Figures and Tables

**Table 1:** Details of SNPs in *IL8* detection.

SNPs	TaqMan SNP genotyping assay ID	Context sequence [VIC/FAM]
*IL8* −845T/C (rs2227532)	C_1842904_10	GCTCTTATGCCTCCACTGGAATTAA[C/T] GTCTTAGTACCACTTGTCTATTCTG

*IL8* −251A/T (rs4073)	C_11748116_10	TTATCTAGAAATAAAAAAGCATACA[A/T] TTGATAATTCACCAAATTGTGGAGC

*IL8* +396T/G (rs222730732)	C_11748168_10	TATTCTGCTTTTATAATTTATACCA[G/T] GTAGCATGCATATATTTAACGTAAA

*IL8* +781C/T (rs2227306)	C_11748169_10	AACTCTAACTCTTTATATAGGAAGT[C/T] GTTCAATGTTGTCAGTTATGACTGT

**Table 2:** Genotype and allele frequencies of *IL8* polymorphisms in control and periodontitis subgroups.

SNP	Genotype	Controls *N* = 156 (%)	CP *N* = 278 (%)	*P* value*	OR (95% CI)	AgP *N* = 58 (%)	*P* value*	OR (95% CI)
*IL8* −251T/A (rs4073)	TT	49 (31.4)	95 (34.2)	—	1.00	18 (31.0)	—	1.00
	TA	78 (50.0)	120 (43.2)	0.37	0.76 (0.47–1.23)	27 (46.6)	0.76	1.08 (0.49–2.37)
	AA	29 (18.6)	63 (22.7)	0.78	1.22 (0.67–2.21)	13 (22.4)	0.67	1.27 (0.47–3.38)
	Allele							
	T	176 (56.4)	310 (55.8)	0.60	1.00	62 (53.4)	0.33	1.00
	A	136 (43.6)	246 (44.2)		0.93 (0.69–1.25)	54 (46.6)		0.89 (0.55–1.44)

*IL8* +396T/G (rs2227307)	TT	54 (34.6)	100 (36.0)	—	1.00	20 (34.5)	—	1.00
	TG	74 (47.4)	118 (42.4)	0.58	0.86 (0.54–1.39)	27 (46.6)	0.74	1.15 (0.54–2.45)
	GG	28 (17.9)	60 (21.6)	0.67	1.28 (0.71–2.31)	11 (19.0)	0.92	1.00 (0.36–2.82)
	Allele							
	T	182 (58.3)	318 (57.2)	0.83	1.00	67 (57.8)	0.89	1.00
	G	130 (41.7)	238 (42.8)		0.91 (0.68–1.23)	49 (42.2)		0.99 (0.61–1.62)

*IL8 *+781C/T (rs2227306)	CC	55 (35.3)	103 (37.1)	—	1.00	20 (34.5)	—	1.00
	CT	76 (48.7)	121 (43.5)	0.51	0.82 (0.52–1.31)	27 (46.6)	0.84	1.10 (0.52–2.34)
	TT	25 (16.0)	54 (19.4)	0.66	1.24 (0.67–2.31)	11 (19.0)	0.66	1.07 (0.38–3.02)
	Allele							
	T	186 (59.6)	327 (58.8)	0.44	1.00	67 (57.8)	0.68	1.00
	C	126 (40.4)	229 (41.2)		1.06 (0.78–1.43)	49 (42.2)		1.05 (0.64–1.70)

*IL8 *−845T/C (rs2227532)	TT	52 (100.0)	120 (98.4)	—	1.00	19 (100.0)	—	1.00
	TC	0 (0.0)	2 (1.6)	0.49	#	0 (0.0)	1.00	#
	CC	0 (0.0)	0 (0.0)	1.00	#	0 (0.0)	1.00	#
	Allele							
	T	104 (100.0)	242 (99.2)	0.49	1.00	38 (100.0)	1.00	1.00
	C	0 (0.0)	2 (0.8)		#	0 (0.0)		#

*Differences between individual haplotypes (between controls and CP or controls and AgP) were analyzed using the Fisher exact test.

OR: odds ratio adjusted for age, sex, and smoking in logistic regression, reference categories designated with an OR of 1.0; CI: confidence interval, ^#^nonapplicable (small numbers); ^#^OR not calculated because of the presence of zero values.

**Table 3:** Distribution of *IL8* haplotypes in the studied groups.

Haplotypes (alleles of *IL8* SNPs)			Controls *N* = 312 (%)	CP *N* = 556 (%)	*P* ^a^	OR (95% CI)	AgP *N* = 116 (%)	*P* ^a^	OR (95% CI)
*IL8* −251T/A (rs4073)	*IL8*+396T/G (rs2227307)	*IL8 *+781C/T (rs2227306)							
T	T	C	162 (51.9)	293 (52.7)	NS	0.99 (0.73–1.32)	62 (53.4)	NS	1.02 (0.63–1.65)
A	G	T	110 (35.3)	212 (38.1)	NS	1.17 (0.86–1.59)	46 (39.7)	NS	1.14 (0.70–1.87)
A	T	T	16 (5.1)	11 (2.0)	**0.018**	**0.34 (0.15–0.78)**	3 (2.6)	NS	0.62 (0.18–2.20)
T	G	C	14 (4.5)	11 (2.0)	**0.048**	**0.41 (0.18–0.97)**	2 (1.7)	NS	0.51 (0.11–2.30)
A	G	C	6 (1.9)	12 (2.2)	NS	1.40 (0.47–4.12)	2 (1.7)	NS	1.22 (0.23–6.35)
A	T	C	4 (1.3)	11 (2.0)	NS	1.79 (0.54–5.90)	1 (0.9)	NS	1.01 (0.11–9.29)
T	T	T	0 (0.0)	3 (0.5)	NS	∗	0 (0.0)	NS	∗
T	G	T	0 (0.0)	3 (0.5)	NS	∗	0 (0.0)	NS	∗

					*P* = NS			*P* = NS	

OR: odds ratio adjusted for age, sex, and smoking in logistic regression CI: confidence intervals.

^a^Differences between individual haplotypes (between controls and CP or controls and AgP) were analyzed using the Fisher exact test.

*OR not calculated because of the presence of zero values.

Bold: significant result.

**Table 4:** Distribution of *IL8* haplotypes (arranged as genotypes) in the studied groups.

Haplotypes (genotypes of *IL8* SNPs) −251T/A, +396T/G, +781C/T/251T/A, +396T/G, and +781C/T	Controls *N* = 156 (%)	CP *N* = 278 (%)	*P* ^a^	OR (95% CI)	AgP *N* = 58 (%)	*P* ^a^	OR (95% CI)
TTC/TTC	42 (26.9)	86 (30.9)	NS	1.18 (0.74–1.87)	17 (29.3)	NS	0.98 (0.46–2.09)
AGT/TTC	63 (40.4)	101 (36.3)	NS	0.80 (0.52–1.23)	24 (41.4)	NS	1.13 (0.57–2.24)
ATT/TTC	6 (3.8)	3 (1.1)	0.08	0.22 (0.05–1.06)	2 (3.4)	NS	1.03 (0.19–5.49)
TGC/TTC	**4 (2.6)**	**1 (0.4)**	**0.046**	**0.09 (0.01–0.96)**	0 (0.0)	NS	**∗**
AGC/TTC	5 (3.2)	6 (2.2)	NS	0.90 (0.24–3.36)	1 (1.7)	NS	0.64 (0.07–6.00)
ATC/TTC	0 (0.0)	6 (2.2)	0.08	∗	1 (1.7)	∗	∗
TTT/TTC	0 (0.0)	3 (1.1)	NS	∗	0 (0.0)	∗	∗
TGT/TTC	0 (0.0)	1 (0.4)	∗	∗	0 (0.0)	∗	∗
AGT/AGT	20 (12.8)	47 (16.9)	NS	1.60 (0.87–2.93)	10 (17.2)	NS	1.12 (0.42–3.04)
ATT/AGT	1 (0.6)	5 (1.8)	NS	2.12 (0.20–22.08)	1 (1.7)	∗	3.36 (0.20–55.97)
AGT/TGC	4 (2.6)	3 (1.1)	NS	0.31 (0.06–1.52)	0 (0.0)	NS	∗
AGT/AGC	1 (0.6)	5 (1.8)	NS	3.28 (0.35–30.98)	1 (1.7)	∗	5.06 (0.29–87.00)
AGT/ATC	1 (0.6)	4 (1.4)	NS	2.52 (0.27–23.83)	0 (0.0)	∗	∗
ATT/ATT	4 (2.6)	1 (0.4)	0.07	0.17 (0.02–1.59)	0 (0.0)	NS	∗
TGC/TGC	3 (1.9)	3 (1.1)	NS	0.71 (0.13–3.82)	1 (1.7)	NS	1.21 (0.12–12.26)
ATT/ATC	1 (0.6)	0 (0.0)	∗	∗	0 (0.0)	∗	∗
ATC/ATC	1 (0.6)	0 (0.0)	∗	∗	0 (0.0)	∗	∗
TGT/TGT	0 (0.0)	1 (0.4)	∗	∗	0 (0.0)	∗	∗
AGC/TGC	0 (0.0)	1 (0.4)	∗	∗	0 (0.0)	∗	∗

			*P* ^b^ = 0.067			*P* ^b^ = NS	

OR: odds ratio adjusted for age, sex, and smoking in logistic regression; CI: confidence intervals.

^a^Differences between individual haplotypes (between controls and CP or controls and AgP) were analyzed using the Fisher exact test.

^b^Differences between individual haplotypes (between controls and CP or controls and AgP) were analyzed using the *χ* ^2^ test.

*OR not calculated because of the presence of zero values.

Bold: significant result.
